# Global Landscape of Glioblastoma Multiforme Management in the Stupp Protocol Era: Systematic Review Protocol

**DOI:** 10.29337/ijsp.148

**Published:** 2021-06-25

**Authors:** Gideon Adegboyega, Ulrick Sidney Kanmounye, Tatjana Petrinic, Ahmad Ozair, Soham Bandyopadhyay, Ashvin Kuri, Yvan Zolo, Katya Marks, Serena Ramjee, Ronnie E. Baticulon, Babar Vaqas, on behalf of InciSion UK Collaborative

**Affiliations:** 1 Queen Mary University of London, Barts and The London School of Medicine and Dentistry, London, United Kingdom; 2 Research Department, Association of Future African Neurosurgeons, Yaounde, Cameroon; 3 Bodleian Health Care Libraries, University of Oxford, Oxfordshire, United Kingdom; 4 Faculty of Medicine, King George’s Medical University, Lucknow, Uttar Pradesh, India; 5 Oxford University Global Surgery Group, Nuffield Department of Surgical Sciences, University of Oxford, Oxfordshire, United Kingdom; 6 Faculty of Health Sciences, University of Buea, Buea, Cameroon; 7 Medical Sciences Division, John Radcliffe Hospital, University of Oxford, OX3 9DU Oxfordshire, Oxford, United Kingdom; 8 Division of Neurosurgery, Department of Neurosciences, Philippine General Hospital, Manila, Philippines; 9 Department of Anatomy, College of Medicine, University of the Philippines Manila, Manila, Philippines; 10 Department of Neurosurgery, Queens Hospital, Romford, United Kingdom; 11 Imperial College London, London, United Kingdom

**Keywords:** glioblastoma multiforme, systematic review protocol, low and middle income countries, healthcare disparities, global neurosurgery, neuro-oncology

## Abstract

**Background:**

Glioblastoma multiforme is the most common and aggressive primary adult brain neoplasm. The current standard of care is maximal safe surgical resection, radiotherapy with concomitant temozolomide, followed by adjuvant temozolomide according to the Stupp protocol. Although the protocol is well adopted in high-income countries (HICs), little is known about its adoption in low- and middle-income countries (LMICs). The aim of this study is to describe a protocol design for a systematic review of published studies outlining the differences in GBM management between HICs and LMICs.

**Methods:**

A systematic review will be conducted. MedLine via Ovid, Embase and Global Index Medicus will be searched from inception to date in order to identify the relevant studies. Adult patients (>18 years) with histologically confirmed primary unifocal GBM will be included. Surgical and chemoradiation management of GBM tumours will be considered. Commentaries, original research, non-peer reviewed pieces, opinion pieces, editorials and case reports will be included.

**Results:**

Primary outcomes will include rates of complications, disability-adjusted life years (DALYs), prognosis, progression-free survival (PFS), overall survival (OS) as well as rate of care abandonment and delay. Secondary outcomes will include the presence of neuro-oncology subspecialty training programs.

**Discussion:**

This systematic review will be the first to compare the current landscape of GBM management in HICs and LMICs, highlighting pertinent themes that may be used to optimise treatment in both financial brackets.

**Systematic Review Registration:**

The protocol has been registered on the International Prospective Register of Systematic Reviews (PROSPERO; registration number: CRD42020215843).

**Highlights:**

- Glioblastoma multiforme (GBM) remains the most common primary adult cerebral neoplasm, with an age-adjusted incidence rate of 3.22 per 100,000 population and a 5-year survival rate of 6.8%
- Despite the well-evidenced efficacy of Stupp protocol, the implementation of this approach bears an institutional and individual financial burden that is particularly notable in low- and middle-income countries (LMICs)
- This systematic review will be the first to compare the current landscape of GBM management in HICs and LMICs, highlighting pertinent themes that may be used to optimise treatment in both financial brackets.

## 1. Introduction

Glioblastoma multiforme (GBM) remains the most common primary adult cerebral neoplasm, with an age-adjusted incidence rate of 3.22 per 100,000 population and a 5-year survival rate of 6.8% [1]. The current standard of care is maximal safe surgical resection, adjuvant radiotherapy and concomitant chemotherapy using temozolomide (TMZ), followed by 6 cycles of TMZ, as proposed by Roger Stupp and colleagues in 2005 [2]. This proposed treatment regimen has been demonstrated to result in a significant survival benefit compared to previous treatment methods [3,4].

Despite the well-evidenced efficacy of Stupp protocol [2], the implementation of this approach bears an institutional and individual financial burden that is particularly notable in low- and middle-income countries (LMICs) [5]. Direct costs arise from the disease itself, relating to the economics of disease management. The introduction of the Stupp regimen increased direct GBM treatment costs eightfold compared to radiotherapy alone, from US$1,200 to US$4,600 per month [6,7]. Additionally, indirect costs such as transportation, meals and housing during the treatment course augment the financial burden [8]. Given the implementation of well-funded public health institutions in high-income countries (HICs) (i.e. the National Health Service in the United Kingdom), the proportion of out-of-pocket (OOP) expenditure is minimal in HICs compared to that seen in LMICs [9]. Additionally, lack of access to radiotherapy services also hinders the ability of LMICs to adequately carry out the gold-standard treatment put forward by Stupp and colleagues [10].

The focus of research to date has centred on intra-country variability in GBM management outcomes [11]. To our knowledge, there is no literature evaluating inter-country variation in provision of optimal management for GBM, hence necessitating the need for a systematic review.

## 2. Objectives and Significance

1. Explore the current standard of GBM management in HICs compared to LMICs with an analysis of the overall survival (OS), progression-free survival (PFS), disability-adjusted life years (DALYs), and complication rates.
2. Assess the rate of abandonment of care (% of patients who are lost to follow up or % not completing treatment) particularly in LMICs.
3. Assess the rates of delay in care on three levels: (1) delay in seeking care, (2) delay in reaching care, and (3) delay in receiving care. Delay in receiving care is further broken down to: (3.1) Delay from diagnosis to surgery, (3.2) Delay from surgery to histologic diagnosis, and (3.3) delay from definitive diagnoses to adjuvant therapy

### Secondary Objective

1. Map out current neuro-oncology subspecialty training and fellowships

## 3. Methods

This protocol has been developed in accordance with the Preferred Reporting Items for Systematic Review and Meta-Analysis Protocols (PRISMA-P) guidelines. The review was registered on the PROSPERO International Prospective Register of Systematic Reviews (Registration Number: CRD42020215843). Protocol amendments will be updated and published alongside the systematic review results (*https://www.crd.york.ac.uk/prospero/display_record.php?RecordID=215843*).

### 3.1. Eligibility Criteria

#### Population

Adult patients (>18 years) with histologically confirmed primary unifocal GBM will be included. Paediatric and adolescent patients, as well as patients with multifocal, recurrent, relapsed, spinal or brainstem GBM will be excluded.

#### Intervention

Evidence-based management of GBM, including surgery, chemotherapy, and radiotherapy, will be considered.

#### Comparisons

This review will consider studies that compare the efficacy of GBM treatment between HICs and LMICs as well as regional variations.

#### Outcomes

Studies that detail rates of complications, DALYs, prognosis, PFS, OS, abandonment of care and treatment delay will be included. Studies reporting health-related quality of life (HRQoL) measures as a primary outcome will be excluded.

#### Type of Study

Commentaries, original research, non-peer reviewed pieces, opinion pieces, editorials and case reports will be included. Conference abstracts, scoping/systematic reviews and book chapters will be excluded.

#### Additional Inclusion Criteria

Studies written in English and French from 2005 to 2020 will be included in the review. Phase III and IV clinical trials and studies outlining the use of genomic sequencing as it relates to overall survival will also be included. Finally, studies focusing on sub-specialty training and fellowships within neuro-oncology will be included.

### 3.2. Information sources

The databases to be searched include: MEDLine via Ovid, Embase and Global Index Medicus.

### 3.3. Search strategy

A search strategy has been developed to identify studies relating to the management of GBM and neuro-oncology training. Synonyms relating to OS, PFS, surgical intervention, chemoradiation therapy, resource limitation, treatment delay, abandonment of care and neuro-oncology education were used (Supplementary Figure 1).

### 3.4. Data management

The data records will first be downloaded from respective databases into EndNote X9. They will then be imported into COVIDENCE Systematic Review Software (Veritas Health Innovation, Melbourne, Australia) where deduplication, title and abstract screening as well as full-text screening will take place. Further data extraction and quality assessment will be carried out on Microsoft Excel (Microsoft, Richmond, Virginia, USA).

### 3.5. Study Selection

A calibration exercise will be carried out before title and abstract screening in order to ensure adequate understanding of the inclusion criteria by study screeners. Deduplication will be undertaken on COVIDENCE Systematic Review Software (Veritas Health Innovation, Melbourne, Australia). Each study will then be screened using title and abstract by 2 independent reviewers. Potentially eligible studies will be further screened for full-text review. Disagreements will be discussed amongst the reviewers and in the case of no resolution an appeal will be made to a third reviewer.

### 3.6. Data Extraction

Full-text screened articles will be exported into a previously-made data extraction proforma on Microsoft Excel (Microsoft, Richmond, Virginia, USA)**. Data will be extracted on (i) study design, (ii) population, (iii) country of origin (iv) tumour characteristics, (v) treatment modalities, (vi) treatment adjuncts (vii) patient outcomes, (viii) abandonment of care, (ix) treatment delay and (x) neuro-oncology training. A short pilot extraction of 5 studies per reviewer will take place in order to assure the reliability of the proforma. Necessary changes will be made upon discussion in order to accurately capture the pertinent themes in the literature.

### 3.7. Risk of Bias Assessment

Two independent reviewers will conduct a risk of bias assessment. The Newcastle-Ottawa Scale (NOS) will be used for observational studies. Cochrane risk of bias 2.0 tool will be used for interventional studies. Conflict resolution will be conducted between the two reviewers, and in the case of no resolution an appeal will be made to a third reviewer.

### 3.8. Data Synthesis

A qualitative analysis of GBM management will be performed. The study design, type and the comparisons detailed in the study will be ascertained. Studies will primarily be dichotomised as either HIC or LMIC. Further sub-distinctions will be made based on geographical location if notable differences emerge in study findings. Pertinent characteristics of the study population will be analysed particularly relating to age, presence of risk-factors and comorbidities, all which contribute to the risk of being diagnosed with GBM and more importantly the subsequent outcomes. Particular features of the tumour will be noted, in order to gauge all the factors that have contributed to tumour prognosis in study participants, such as those related to tumour biology and tumour recurrence. The extent of surgical management for these tumours will be highlighted; a particular note will be taken as to whether these studies adhere to the recommended management of GBM proposed by Stupp et al [2], and reasons for falling short of this management protocol will be noted. Additionally, non-pharmacological therapies used in GBM treatment will also be determined, in order to assess the overall effectiveness of rehabilitation, education and other non-pharmacological therapies in the absence of adjuvant therapy, particularly in developing countries.

Four core treatment outcomes will be described: OS, PFS, DALYs and complication rates. An analysis of the treatment outcomes will enable a comparison of the treatment techniques between HICs and LMICs, as well as between two regions in the same financial bracket. The abandonment of care in studies will be assessed and reasons for abandonment will be outlined. Training programs in the regions of each study will be analysed, with a specific focus on the exposure to cases, the number of training hours and an analysis of any comments pertaining to the quality of these programs.

Finally, the delay in management of care will also be scrutinised using the three-delay model: delay in seeking, reaching, and getting care. Delay in care can be seen in a number of ways which can increase risk of morbidity and mortality. The number of these delays will be determined and possible solutions in order to correct these delays will be suggested.

## 4. Ethics and Dissemination

This study will exclusively involve secondary data collection and no human participants will be involved in the design or dissemination of this research, hence ethical approval was not required. The results from this study will be disseminated through a peer-reviewed journal.

## 5. Limitations

There is an extensive amount of literature published in Mandarin Chinese, Russian, and Portugese that will not be addressed by this review.

## 6. Conclusion

The proposed systematic review aims to detail the current landscape of GBM management worldwide, highlighting parallels and differences among countries in different income groups. This novel work will allow for a better understanding of the current situation in the developing world, where-in important lessons can be drawn from the robust learning environment in HICs, and vice-versa from the “out-of-box” thinking that is common in regions with limited resources. Despite the poor survival rate of patients with GBM, there are still pertinent lessons to be learnt to improve patient outcomes and the quality of care.

## Additional Files

The additional file for this article can be found as follows:

10.29337/ijsp.148.s1 Supplementary Figure 1. MEDLine Search Strategy.

## Amendments

Any amendments to this protocol will be prospectively updated on the PROSPERO International Prospective Register of Systematic Reviews.

## Abbreviations

GBM: Glioblastoma Multiforme

HICS: High-income countries

LMICS: Low and middle-income countries

TMZ: Temozolomide

OOP: Out-of-pocket

OS: Overall survival

PFS: Progression-free survival

DALYS: Disability-adjusted life-years

PRISMA-P: Preferred Reporting Items for Systematic Review and Meta-Analysis Protocols
