# Supplementary material for: Global Landscape of Glioblastoma Multiforme Management in the Stupp Protocol Era: Systematic Review Protocol
Source: Int J Surg Protoc. 2021 Jun 25;25(1):108–13. doi: 10.29337/ijsp.148 (PMC8231457; doi:10.29337/ijsp.148)
Supplement: Supplementary Figure 1. — MEDLine Search Strategy. [file ijsp-25-1-148-s1.pdf]

Supplementary Figures

Supplementary Figure 1: MEDLine Search Strategy

1. GLIOBLASTOMA/
2. "glioblastoma*".ab,kw,ti.
3. "astrocytoma*".ab,kw,ti.
4. glioma*.ab,kw,ti.
5. 1 or 2 or 3 or 4
6. (multiform* or GBM* or "giant cell" or "grade IV" or "grade 4" or "high grade").ab,kw,ti.
7. 5 and 6
8. limit 7 to ("all infant (birth to 23 months)" or "all child (0 to 18 years)")
9. (pediatric* or paediatric* or child or children or infant* or neonat* or newborn* or adolescen*).ti.
10. 7 and 9
11. 8 or 10
12. 7 not 11
13. limit 12 to animals
14. 12 not 13
15. RADIOTHERAPY/
16. RADIOTHERAPY, ADJUVANT/
17. RADIOSURGERY/
18. CRANIAL IRRADIATION/
19. (radiotherap* or radiotherapy or irradiation or "radiation therapy").ab,kw,ti.
20. CHEMOTHERAPY/
21. CHEMOTHERAPY, ADJUVANT/
22. CHEMORADIOTHERAPY/
23. TEMOZOLOMIDE/
24. (chemotherap* or chemotherapy* or temozolomide).ab,kw,ti.
25. 15 or 16 or 17 or 18 or 19 or 20 or 21 or 22 or 23 or 24
26. 14 and 25
27. TREATMENT OUTCOME/
28. TREATMENT FAILURE/
29. SURVIVAL RATE/
30. PROGRESSION-FREE SURVIVAL/
31. INTRAOPERATIVE COMPLICATIONS/
32. POSTOPERATIVE COMPLICATIONS/
33. (outcome* or survival or survivor* or complication*).ab,kw,ti.
34. ("disability adjusted" or DALY*).ab,kw,ti.

35. (haemorrhag* or hemorrhag* or "major bleed*").ab,kw,ti.

36. (stroke* or "cerebrovascular event*" or "cerebrovascular accident*").ab,kw,ti.

37. (dipopia* or "double vision").ab,kw,ti.

38. hemiparesis.ab,kw,ti.

39. "sensory deficit*".ab,kw,ti.

40. "cranial neuropath*".ab,kw,ti.

41. "cognitive decline".ab,kw,ti.

42. "seizure* ".ab,kw,ti.

43. "aphasia".ab,kw,ti.

44. 27 or 28 or 29 or 30 or 31 or 32 or 33 or 34 or 35 or 36 or 37 or 38 or 39 or 40 or 41 or 42 or 43

45. 26 and 44

46. NEUROSURGEON/ or NEUROSURGERY/

47. ONCOLOGIST/ or SURGICAL ONCOLOGY/ or MEDICAL ONCOLOGY/

48. ("neurosurgical oncolog*" or "neuro oncolog*").ab,kw,ti.

49. 46 or 47 or 48

50. MEDICAL EDUCATION, GRADUATE/

51. INTERNSHIP/ or RESIDENCY/

52. SPECIALIZATION/

53. (training or trained or trainee* or education or teaching or curricul* or specializing or specialising).ab,kw,ti.

54. 50 or 51 or 52 or 53

55. 49 and 54

56. 45 or 55
